# Docosahexaenoic Acid Inhibits Cell Proliferation through a Suppression of c-Myc Protein in Pancreatic Ductal Adenocarcinoma Cells

**DOI:** 10.3390/antiox10111721

**Published:** 2021-10-28

**Authors:** Jia-Ning Syu, Der-Yen Lee, Hung-Chang Hung, Chia-Ying Li, Hung-Yu Lin, En-Pei Isabel Chiang, Yi-Heng Chen, Shu-Ming Huang, Feng-Yao Tang

**Affiliations:** 1 Biomedical Science Laboratory, Department of Nutrition, China Medical University, Taichung 40604, Taiwan; mine800815@gmail.com; 2 Graduate Institute of Integrated Medicine, China Medical University, Taichung 404333, Taiwan; deryen.lee@mail.cmu.edu.tw; 3 Department of Internal Medicine, Nantou Hospital, Ministry of Health and Welfare, Nantou City 540, Taiwan; h550327@yahoo.com.tw; 4 Department of Surgery, Show Chwan Memorial Hospital, Changhua 500, Taiwan; b86401115@ntu.edu.tw (C.-Y.L.); eeepangting@hotmail.com (Y.-H.C.); 5 Research Assistant Center, Show Chwan Memorial Hospital, Changhua 500, Taiwan; linhungyu700218@gmail.com; 6 Department of Food Science and Biotechnology, National Chung Hsing University, Taichung 402, Taiwan; chiangisabel@email.nchu.edu.tw; 7 Innovation and Development Center of Sustainable Agriculture (IDCSA), National Chung Hsing University, Taichung 402, Taiwan; 8 Department of Nutrition, Nantou Hospital, Ministry of Health and Welfare, Nantou City 540, Taiwan; alice926112@gmail.com; 9 Department of Nutrition, Master Program of Biomedical Nutrition, Hungkuang University, Taichung 433304, Taiwan

**Keywords:** DHA, STAT3, CAMKII, c-Myc, oxidative stress, cell apoptosis, nucleotide synthesis, pancreatic ductal adenocarcinoma

## Abstract

Treatment of pancreatic cancer by inhibiting the aberrant activation of the survival signaling pathways has received considerable attention. We investigated the probable action of DHA on the suppression of cell proliferation in human pancreatic ductal adenocarcinoma (PDAC) cells. Our results demonstrated that DHA dose-dependently inhibited cell proliferation through an induction of cell cycle arrest in human PDAC cells. DHA suppressed the expression of phosphorylated-Rb (p-Rb), cyclin D1, cyclin E, cyclin A, E2F1 and c-Myc proteins. Blocking the activation of STAT3 signaling pathway led to an inactivation of CAMKII and increased phosphorylation of c-Myc (T58) protein accompanied with decreased expression of c-Myc protein. Treatment of DHA effectively inhibited cell survival through decreased phosphorylation levels of EGFR, STAT3 and CAMKII proteins. The mechanisms of action were associated with increased phosphorylation levels of c-Myc (T58) and instability of c-Myc proteins. DHA inhibited cell survival through an increased GSSG/GSH ratio and oxidative stress level in HPAF-II cells. DHA induced cell apoptosis through increased expression of Bax, c-caspase 3 and c-PARP proteins in HPAF-II cells. Moreover, treatment of DHA significantly inhibited nucleotide synthesis. In conclusion, DHA might significantly suppress the proliferation of PDAC cells and therefore have potential as an anti-cancer therapeutic agent.

## 1. Introduction

Pancreatic cancer is one of the leading causes of cancer mortality in many countries [1]. Around 85% of pancreatic cancers belong to the adenocarcinoma subtype [2]. Patients with pancreatic ductal adenocarcinoma (PDAC) have a five-year survival rate of only 7% [2]. More than 90% of PDAC patients have mutationally activated *Kras* oncogene [3]. The reprogrammed metabolism and aberrant nucleotide synthesis were driven by *Kras* mutation in PDAC cells [4,5]. PDAC cells are dependent on glucose to maintain their metabolisms for proliferation and regulate anti-apoptotic escape [4].

Recent studies also suggest that the prevalence of PDAC is strongly associated with diabetes mellitus (DM) [6,7]. Results from Pancreatic Cancer Cohort (PanScan) Consortium indicate that two to eight years duration of DM would increase risk of PDAC by about 80% [8]. Another study indicated that hyperglycemia induced tumor progression through increased levels of phosphorylated STAT3 and Myc proteins in KRAS-mutant pancreatic cancer in vivo [9]. This evidence suggested that high glucose levels could have a significant impact on the progression and promotion of PDAC cells.

Receptor tyrosine kinase (RTK) such as epidermal growth factor (EGFR) plays an important role in determining survival and progression of cancer cells [10,11]. The hyper-phosphorylation of EGFR would lead to the activation of survival signaling pathways and enhance cell progression [10]. The hyper-phosphorylation of EGFR also enhances the activation of Janus Kinase (JAK)/activation of Signal Transducer and Activator Transcription 3 (STAT3) signaling and cell survival [12,13]. A recent study indicated that the activation of STAT3 signaling plays an important role in therapeutic resistance to RAS inhibition in PDAC [14]. Blockade of STAT3 also impaired the expression of c-Myc and inhibited tumor growth [15]. Therefore, STAT3 signaling and downstream c-Myc protein could be probable targets to the treatment of PDAC cells.

Deregulation of c-Myc signaling has been observed in various human cancers [16]. The stability of c-Myc is mediated by two phosphorylation positions at serine 62 (Ser62) and threonine 58 (Thr58) [16,17]. Phosphorylation of Ser62 promotes c-Myc protein stability, while subsequent phosphorylation of Thr 58 by glycogen synthase kinase-3β (GSK3-β) triggers Ser62 dephosphorylation, resulting in c-Myc degradation and turnover [17,18,19]. Cell cycle progression through the different phases is under the control of cyclin, the regulatory subunit, and cyclin-dependent protein kinase (CDK) [20]. Stable c-Myc targets at the cyclin genes and induces CDK activity, tightly controlling cell cycle progression and nucleotide synthesis [20]. A recent study demonstrated that CAMK-II protein enhances in the stability of c-Myc protein by phosphorylating it at Ser62, thereby allowing nuclear translocation of c-Myc and facilitating cell survival [21]. However, the correlation among STAT3/ CAMKII signaling pathways and c-Myc protein has not yet been well studied in human PDAC cells.

Despite the remarkable treatments of PDAC using molecular targeted agents, the challenge for cancer treatment stills concerns acquisition of resistance. For example, activation of compensatory kinase, or modification of signaling pathway allows cancer cell survival during target inhibition [14]. Epidemiologic studies indicate that consumption of fish oil has a significant reduction in pancreatic cancer risk [22]. However, the literature remains unclear regarding the anti-cancer actions of DHA in human PDAC cells. Our previous study indicated that DHA, one of N-3 polyunsaturated fatty acids (PUFAs), could inhibit the proliferation of human PDAC cells in vitro and in vivo [23]. This study aimed to investigate the inhibitory effects of DHA and novel mechanisms on the suppression of these candidate molecules including STAT3/CAMKII/c-Myc proteins in PDAC cells upon high glucose stimulation.

## 2. Materials and Methods

### 2.1. Reagents, Chemicals and Antibodies

Human PDAC HPAF-II (ATCC^®^ CRL-1997™), CFPAC-1 (ATCC^®^ CRL-1918™) and MIA PaCa-2 (ATCC^®^ CRL-1420™) cell lines were acquired from American Type Culture Collection (Manassas, VA, USA) and provided by the laboratory of Dr. Wen-Hwa Lee of Genomics Research Center, Academia Sinica (Taiwan, China). Human PDAC HPAF-II, CFPAC-1 and MIA PaCa-2 cells were cultured in 10% fetal bovine serum (FBS) Dulbecco’s Modified Eagle’s Medium (DMEM). In this study, human PDAC cells were treated with DHA (at concentrations of 25, 50, 100 and 150 μM).

The following antibodies were obtained from Cell Signaling Technology, Inc. (Danvers, MA): anti-phospho-Rb Ser795(p-Rb S795), anti-cyclin E, anti-E2F1, anti-c-Myc, anti-phospho-EGFR Tyr1068 (p-EGFR Y1068), anti-phospho-STAT3 Tyr705 (p-STAT Y705), anti-total-STAT3 (t-STAT3), anti-phospho-CAMKII Thr286 (p-CAMKII T286), anti-Bax, anti-cleaved- caspase-3, anti-cleaved-PARP, anti-actin and anti-Lamin A. The following antibodies were purchased from Santa Cruz Biotechnology Inc. (Dallas, TX, USA): anti-Rb, anti-cyclin D1, anti-cyclin A, anti-Bcl-2. Anti-phospho-c-Myc Thr58 (p-c-Myc T58) antibody was obtained from Elabscience (Waltham, MA, USA). DHA, lapatinib, ruxolitinib, MG-132, Z-VAD-FMK, GSH (reduced form of glutathione), NAC (N-acetyl-cysteine) and ethanol were obtained from Sigma (St. Louis, MO, USA). The nuclei and cytoplasm protein extract kit were purchased from Pierce Biotechnology Inc. (Lackford, IL, USA). Propidium Iodine (PI) was acquired from BD Biosciences Inc. (Franklin Lakes, NJ, USA). Dulbecco’s Modified Eagle’s Medium (DMEM) and fetal bovine serum (FBS) were purchased from Invitrogen Inc. (Carlsbad, CA, USA).

### 2.2. Cell Culture and Treatment of DHA

Human PDAC cells were cultured and grown in DMEM media supplemented with 10% FBS, 2 mM L-glutamine and 1.5 g/L sodium bicarbonate.

PDAC cells were incubated with various concentrations (25, 50, 100 and 150 μM) of the DHA. For efficient treatment of PDAC cells, DHA was dissolved in ethanol and incorporated into FBS and mixed with the medium. In control groups, cells were incubated with an equivalent volume of solvent ethanol (final concentration: 0.05% *v*/*v*) as a carrier vehicle.

### 2.3. Assessment of Cell Proliferation

Detection of cell proliferation analysis was performed using the MTT assay according to the previous protocol [24]. Human PDAC cells including HPAF-II, CFPAC-1 and MIA PaCa-2 (2 × 10^4^ cells/well) were seeded and grown in a 24-well plate. These PDAC cells were cultured in media containing DHA (at concentrations of 0, 25, 50, 100 and 150 μM). Cell proliferation assay was performed in a triplicate test. At the end of experiments, cultured media were removed from each plate and MTT reagent (3-[4,5-dimethhylthiaoly]-2,5-diphenyltetrazolium bromide) at a working concentration of 0.5 mg/mL was added to each well. After a 2-hr incubation, the MTT solution was removed, and isopropanol was added to each well. These culture plates were vibrated for 10 min to dissolve the precipitation. Optical density was measured at wavelength of 570 nm with a plate reader.

### 2.4. Analysis of Cell Cycle Distribution

Human PDAC HPAF-II cells (1 × 10^6^ cells/well) were cultured in 3 cm culture plates. Cells were synchronized to the same cell cycle stage by culturing in DMEM media with 0.05% FBS before initiating the experiment. To measure the effects of DHA on the cell cycle distribution, human PDAC HPAF-II cells were treated with DHA (0, 25, 50, 100 and 150 μM) for another 24 h according to previous reference [25,26]. Cells were detached with a trypsin/EDTA solution and later mixed with the binding buffer (1 × 10^5^ cells/mL). Suspended HPAF-II cells were treated with 0.5 mg/mL RNases for the removal of RNA, 0.1% Triton X-100 for permeabilization and then stained with 4 μg/mL PI in the dark and were analyzed using a FACSCanto flow cytometry system (BD Biosciences Inc., Franklin Lakes, NJ, USA). The degree of PI-staining in human PDAC HPAF-II cells was measured with the help of an accessory ModFit LT version 3.2 Software (Verity Software House, Topsham, ME, USA). 

### 2.5. Annexin-V-Propidium Iodide-Binding Assay

To determine whether DHA affected the apoptosis of PDAC HPAF-II cells, the apoptotic level of HPAF-II cells was detected by the Annexin V-FITC Apoptosis Detection Kit according to the manufacturer’s instruction and previous reference [26]. Briefly, after incubation with DHA at different concentrations (0, 25, 50, 100 and 150 μM), 5 × 10^6^ isolated HPAF-II cells were resuspended in 500-μL-binding buffer. FITC-annexin V (5 μL) and propidium iodide (PI, 5 μL) working solution were added and then cells incubated at room temperature for 5 min. At the end of the incubation period, cells were analyzed by flow cytometry, the annexin V-FITC binding was analyzed by FITC signal detector and PI staining by phycoerythrin emission signal detector.

### 2.6. Protein Extraction and Western Blotting Analysis

Protein extraction (cytoplasmic and nuclear proteins) of human HPAF-II cells were prepared using the Nuclear Protein Extract Reagent Kit containing inhibitors against protease and phosphatase. To remove the cell debris, cell lysates were centrifuged at 12,000× *g* for 10 min. The upper phase of supernatants was kept as a cytoplasmic extract. The remaining precipitation was kept as a nuclear extract. Cellular proteins (60 μg) fractionated by 10% SDS-PAGE were transferred to a PVDF membrane and detected with anti-p-STAT3(Y705) monoclonal antibody. The remaining proteins in the cell lysates were measured using antibodies of anti- p-Rb(S795), anti-Rb, anti-cyclin D1, anti-cyclin E, anti-cyclin A, anti-E2F1, anti-c-Myc, anti- p-c-Myc (T58), anti- p-EGFR(Y1068), anti-t-STAT3, anti-p-CAMKII(T286), anti-Bax, anti-Bcl-2, anti-cleaved-caspase-3 (c-caspase 3), and anti-cleaved-PARP (c-PARP). These blots were probed with internal control antibodies against actin or lamin A proteins.

### 2.7. Measurement of Intracellular Reactive Oxygen Species (ROS)

Human PDAC HPAF-II cells were cultured in 6-well plates and treated with DHA for 24 h. The intracellular ROS levels were measured according to the previous study [27]. Briefly, DHA- treated HPAF-II cells were incubated with 2′,7′-dichlorofluorescin diacetate (DCFH-DA) at a final concentration of 10 µM in serum free medium for 30 min. After the incubation, cells were collected and resuspended with 300 μL PBS for the analysis of ROS by using BD FACS Canto flow cytometry (BD Biosciences Inc., Franklin Lakes, NJ). The fluorescence intensity derived from 2′,7′-dichlorofluorescein (DCF) represented the intracellular ROS levels.

### 2.8. Analysis of Thiol Compounds and Nucleotides

The protocol for analysis of thiol compounds and nucleotides was based on the previous study [23]. For the analysis of thiol compounds, metabolite samples were mixed with the reaction solution, containing 20 mM sodium carbonate (pH 9.5) and 0.2 mM ^13^C_6_-2-iodoacetaniline (^13^C_6_-2-IAN). The reaction mixture was incubated at 70 °C for 2 h and later terminated by the addition of 50 μL of 2% formic acid. Samples were centrifuged at 14,000 rpm for 10 min and the supernatants were subjected to analysis using the Vion IMS QTOF system.

For the analysis of nucleotides, cellular metabolite samples were mixed with the reaction solution, containing 30 μL of ddH_2_O, 5 μL of 0.3 M of aniline dissolved in HCl and 5 μ of 20 mg/mL N-(3-dimethylaminopropyl)- ethylcarbodiimide hydrochloride (EDC). The mixture was centrifuged at 14,000 rpm for 5 s, vortexed and incubated at room temperature for 2 h. The reaction was terminated by adding 5 μL of 10% ammonium hydroxide followed by a 30 min incubation at room temperature. Then the samples were centrifuged at 14,000 rpm for 10 min and the supernatants were subjected to analysis using the Vion IMS QTOF system.

We briefly evaluated the process of using Liquid chromatography(LC)-electrospray ionization (ESI)-mass spectrometry(MS) analysis done in our study. The LC-ESI-MS system consisted of an ultra-performance liquid chromatography (UPLC) system (ACQUITY UPLC I-Class, Waters) and an ESI/APCI source of 4 kDa quadrupole time-of-flight (TOF) mass spectrometer (Waters VION, Waters). The flow rate was set to 0.2 mL/min with a column temperature at 35 °C. Separation was performed by using reversed-phase liquid chromatography (RPLC) technique on a BEH C18 column (2.1 × 100 mm, Walters) with 5 μL sample injection. The elution started initially from 99% mobile phase A (ultrapure water + 0.1% formic acid) and 1% mobile phase B (100% methanol + 0.1% formic acid), held at 1% B for 0.5 min, raised to 90% B in 5.5 min, held at 90% B for 1 min, and then lowered to 1% B in 1 min. The column was equilibrated by pumping 1% B for 4 min. LC-ESI-MS chromatogram was acquired by ESI+ mode which was kept under following conditions: capillary voltage kept at 2.5 kV, source temperature maintained at 100 °C, desolvation temperature regulated at 250 °C, cone gas maintained at 10 L/h, desolvation gas maintained at 600 L/h, and an acquisition by MSE mode with a range of *m/z* 100–1000 and a 0.5 s scan time. The acquired data was processed by UNIFI software (Waters) with an illustrated chromatogram and summarized in an integrated area of signals.

### 2.9. Biostatistical Analysis

A statistical analysis was used to determine the significant difference in the cell viability between the control subgroup and the experimental subgroups of PDAC cells using SYSTAT software. We used one-way ANOVA model to confirm a significant difference in cell viability which required an exclusion of null difference between the mean values originated from different subgroups at the *p* = 0.05 level. A Duncan’s multiple range test was applied to evaluate differences among these subgroups.

## 3. Results

### 3.1. DHA Inhibited the Proliferation of Human PDAC HPAF-II Cells in Association with Downregulation of Cyclin D1 and c-Myc Proteins

**Figure 1:**
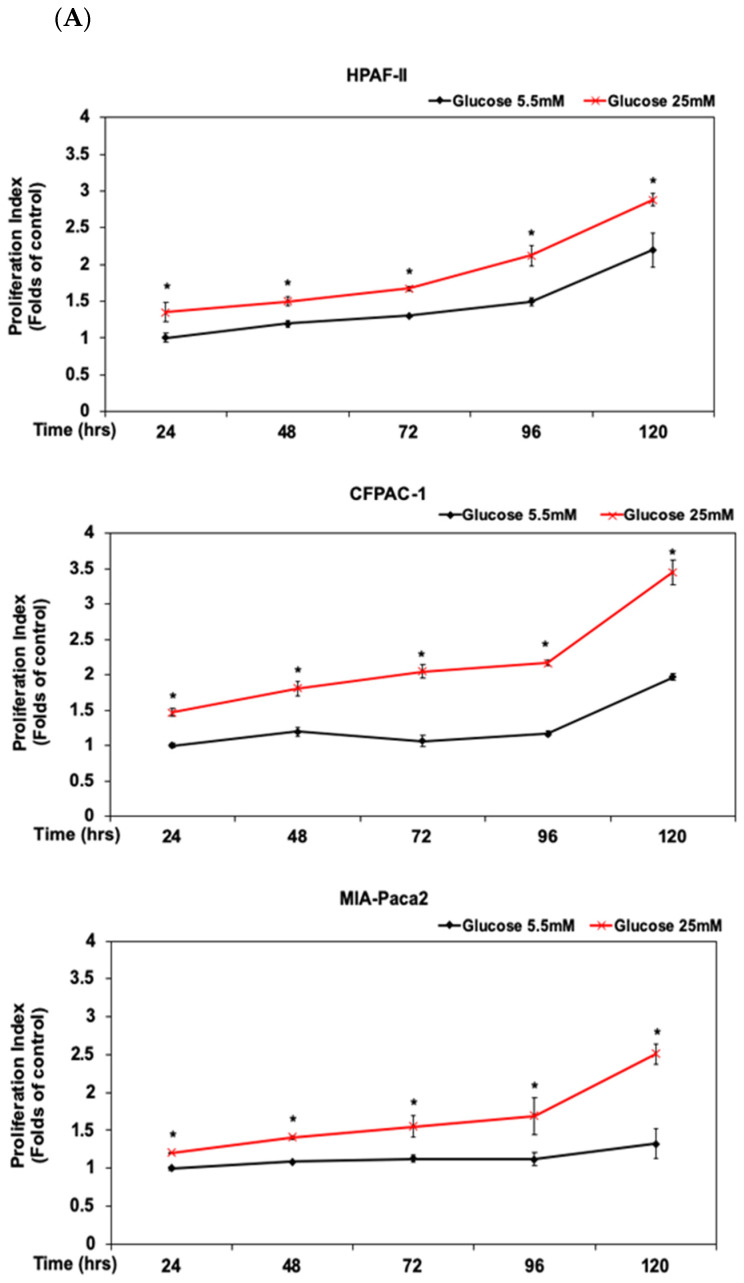

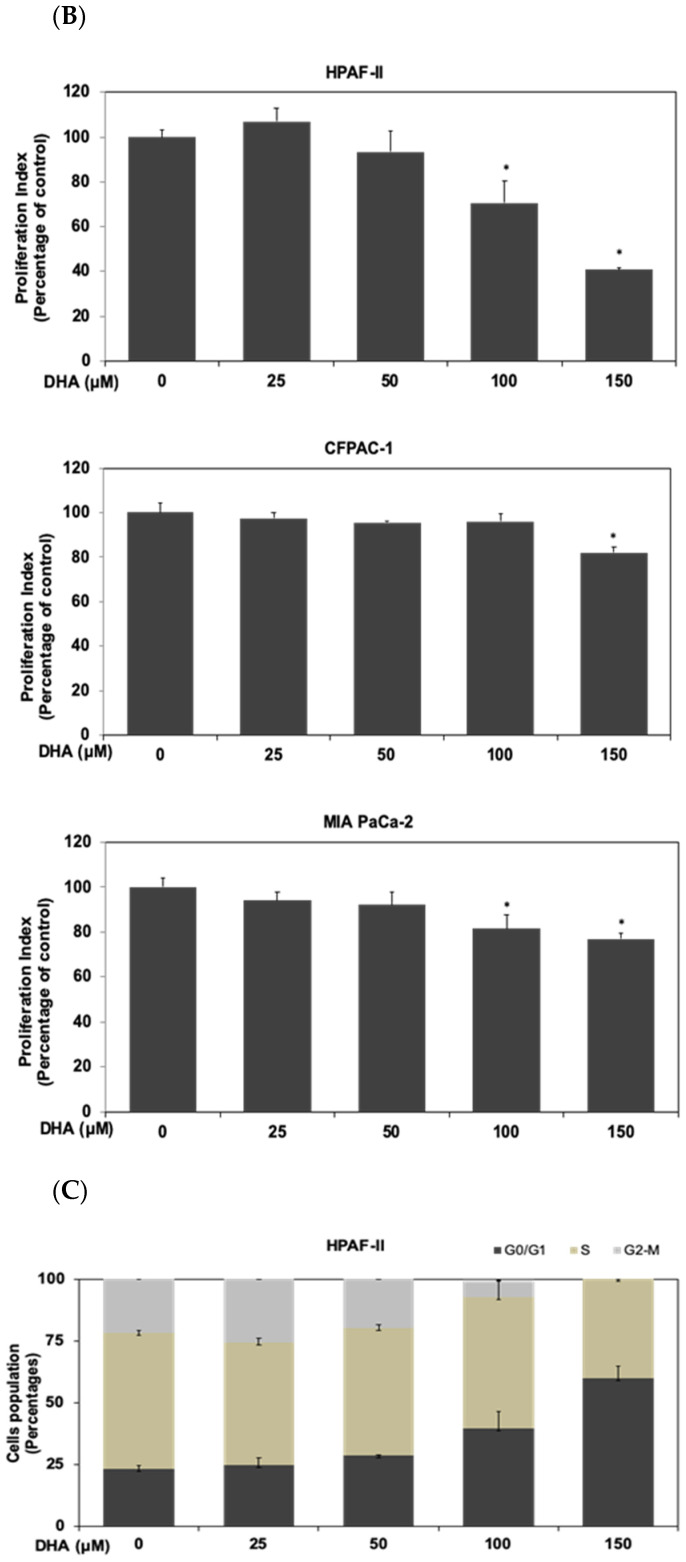

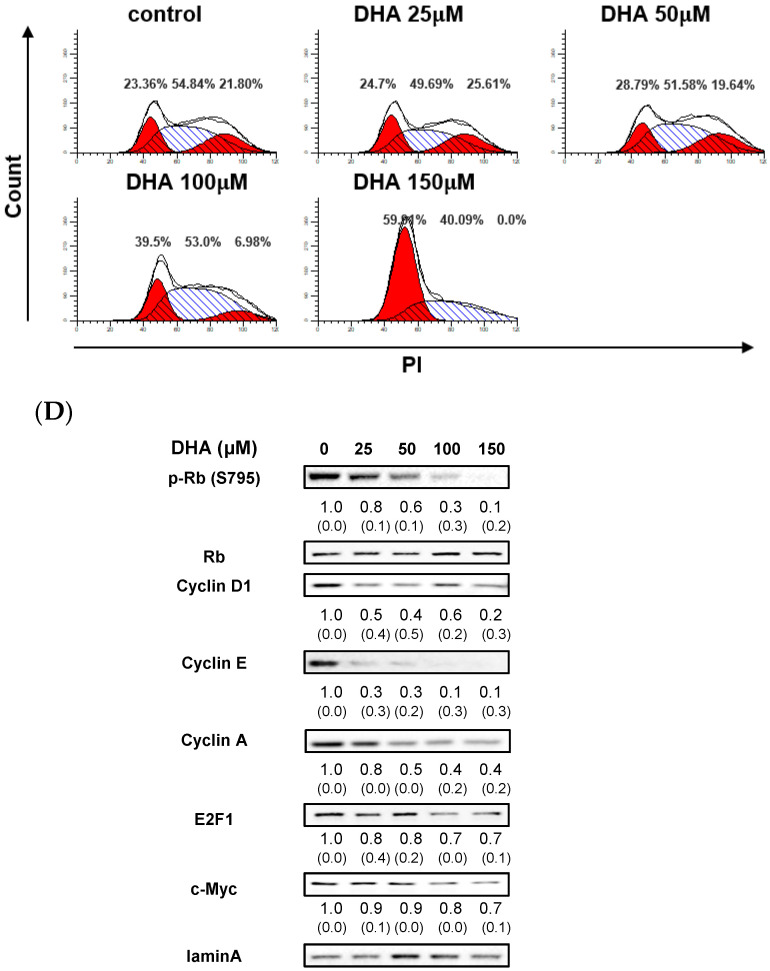
DHA inhibited the proliferation of human PDAC HPAF-II cells in association with downregulation of cyclin D1 and c-Myc proteins. (**A**) Human PDAC HPAF-II, CFPAC-1 and MIA PaCa-2 cells were cultured in DMEM with different concentrations of glucose (5.5 and 25 mM) for various time points (24, 48, 72, 96 and 120 h). Cell survival was analyzed using MTT assay as described in Materials and Methods Section. Proliferation index, in comparison with the control subgroup (at a concentration of 5.5 mM glucose), is expressed as the mean ± SD (standard deviation) of three independent experiments. An asterisk (*) represent statistically significant differences in comparison with the control subgroup at the same time point (*p* < 0.05). (**B**) Human PDAC HPAF-II, CFPAC-1 and MIA PaCa-2 cells were treated with DHA in DMEM containing 25 mM glucose for 24 h, respectively. At the end of experiment, cell survival was analyzed using MTT assay as described previously. An asterisk (*) represent statistically significant differences in comparison with the control subgroup at the same time point (*p* < 0.05). (**C**) Human PDAC HPAF-II cells were treated with DHA in DMEM containing 25 mM glucose for 24 h. Measurement of the cell population at different cell cycle phases was performed using flow cytometry analysis, as described in Materials and Methods Section. (**D**) Western blotting analysis of nuclear proteins (at 24 h time point) was performed using antibodies against p-Rb (S795), Rb (internal control for p-Rb), cyclin D1, cyclin E, cyclin A, E2F1, c-Myc and lamin A. Lamin A was used as the internal control for cyclin D1, cyclin E, cyclin A, E2F1, c-Myc. Band intensities represent as the mean ± SD (within parenthesis) for the amount of each nuclear protein in comparison with corresponding control protein.

Human PDAC HPAF-II, CFPAC-1 and MIA PaCa-2 cells were treated with glucose at different concentrations (5.5 and 25 mM) for the analysis of cell proliferation levels at different time points (24, 48, 72, 96 and 120 h), respectively (Figure 1A). Our results showed that high concentration of glucose (HG) (25 mM) significantly induces the proliferation of human PDAC cells in a dose- dependent manner (*p* < 0.05). Glucose at a dosage of 25 mM significantly induces the proliferation of HPAF-II cells up to 1.4 folds in comparison with the control subgroup (5.5 mM of glucose) at 24 h time point (Figure 1A). Similar findings were also observed in CFPAC-1 and MIA PaCa-2 cells upon the stimulation of HG (25 mM) (Figure 1A). These results suggested that HG condition, at a concentration of 25 mM, significantly enhanced the proliferation of PDAC cells in vitro (*p* < 0.05). Previous studies indicated that consumption of fish oil abundant with N-3 polyunsaturated fatty acids (N-3 PUFAs) such as DHA is correlated with a low prevalence of pancreatic cancer [22]. Therefore, we selected HPAF-II cells as the major cell model and examined whether treatment of DHA could inhibit the proliferation of human PDAC cells under the stimulation of HG in this study. In comparison with the control subgroup, DHA dose-dependently inhibited the proliferation of human PDAC HPAF-II, CFPAC-1 and MIA PaCa-2 cells upon the stimulation of HG at 24 h time point (*p* < 0.05) (Figure 1B). The inhibitory concentration 50% (IC_50_) of DHA for HPAF-II, CFPAC-1 and MIA PaCa-2 cells were 80.7 μM, 400 μM and 310 μM, respectively(Supplementary Table S1). We further investigated whether DHA could inhibit cell proliferation through a modulation of cell cycle progression. As shown in Figure 1C, treatment of DHA induced cell cycle arrest at G_0_/G_1_ phase in HPAF-II cells upon the stimulation of HG. These results suggested that treatment of DHA significantly inhibited the proliferation of HPAF-II cells even under the stimulation of HG. To study the underlying mechanisms of action, we further investigated whether treatment of DHA could suppress the expression of cell cycle regulatory proteins in human HPAF-II cells. As shown in Figure 1D, DHA effectively inhibited the expression of phosphorylated Rb (p-Rb), cyclin D1, cyclin E, cyclin A, E2F1 and c-Myc proteins. These results suggested that DHA could act as an effective agent to suppress cell proliferation through downregulation of cell cycle regulatory proteins upon the stimulation of HG in human HPAF-II cells.

### 3.2. Activation of STAT3/CAMKII Signaling Pathway Is Involved in the Phosphorylation and Expression of c-Myc Protein in HPAF-II Cells

**Figure 2:**
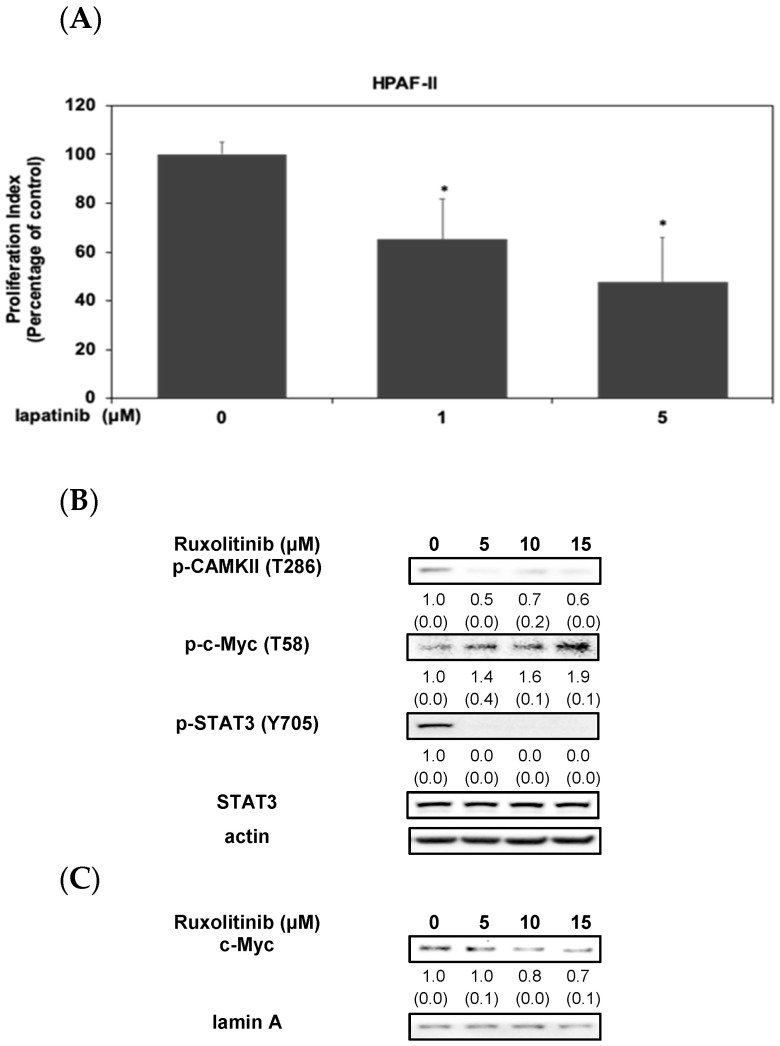
Activation of STAT3/CAMKII signaling pathway is involved in the phosphorylation and expression of c-Myc protein in HPAF-II cells. (**A**) Human PDAC HPAF-II cells were treated with lapatinib (at concentrations of 1 and 5 μM), a specific inhibitor of EGFR, in 10% FBS DMEM containing 25 mM glucose for 24 h. Cell proliferation was analyzed using MTT assay as described in Materials and Methods Section. Statistical significance is expressed as the mean ± SD (standard deviation) of three independent experiments. An asterisk (*) represent statistically significant differences in comparison with the control subgroup (*p* < 0.05). Human PDAC HPAF-II cells were treated with ruxolitinib (at concentrations of 5, 10 and 15 μM), a specific inhibitor of JAK/STAT3 signaling pathway, in 10% FBS DMEM for 24 h. Western blotting analysis of cytoplasmic (**B**) and nuclear proteins (**C**) (at 24 h time point) was performed using antibodies against p-CAMKII (T286), p-c-Myc (T58), p-STAT3 (Y705), STAT3, c-Myc, actin and lamin A. Band intensities represent as the mean ± SD (within parenthesis) for the amount of cytoplasmic and nuclear proteins in comparison with corresponding control proteins, including STAT3 (internal control for p- STAT3), actin and lamin A, respectively.

Our results indicated that DHA worked as an effective agent to suppress the proliferation of human PDAC HPAF-II cells (Figure 1). Thus, we further investigated the underlying anti-cancer mechanism of DHA. As shown in Figure 2A, treatment of lapatinib (a specific inhibitor of EGFR) significantly inhibited the proliferation of PDAC cells upon HG stimulation. These results indicated that HG-mediated cell proliferation of human PDAC HPAF-II cells was, in part, associated with the activation of EGFR protein. Therefore, we further examined the probable signaling pathways. As shown in Figure 2B, treatment of ruxolitinib, a specific JAK/STAT inhibitor, effectively inhibited expression of c-myc protein and the phosphorylation levels of CAMKII and STAT3 in HPAF-II cells. Interestingly, ruxolitinib significantly induced the phosphorylation of c-Myc protein (at Thr 58 residue). These results indicated that the inactivation of STAT3 signaling protein could lead to an instability and decreased expression of c-myc protein in HPAF-II cells.

### 3.3. DHA Induced the Proteasomal Degradation and Instability of c-Myc Protein in HPAF-II Cells

**Figure 3:**
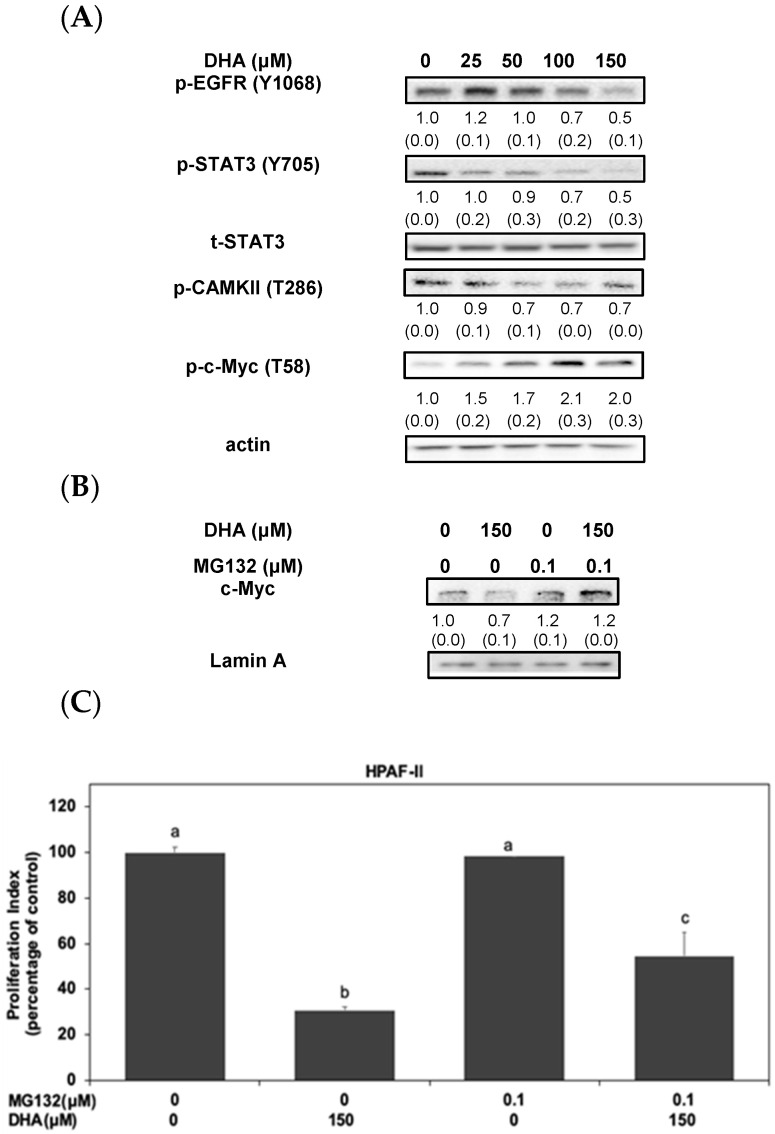
DHA induced the proteasomal degradation and instability of c-Myc protein in HPAF-II cells. (**A**) Human PDAC HPAF-II cells were treated with DHA in 10% FBS DMEM for 24 h. Western blotting analysis of cytoplasmic and nuclear proteins (at 24 h time point) was performed using antibodies against p-EGFR (Y1068), p-STAT3 (Y705), t-STAT3, p-CAMKII (T286), p-c-Myc (T58) and actin. Band intensities are represented as the mean ± SD (within parenthesis) for the amount of each protein in comparison with internal control actin. (**B**) Human PDAC HPAF-II cells were treated with DHA (150 μM) in 10% FBS DMEM in the presence or absence of MG-132 in 10% FBS DMEM for 24 h. Western blotting analysis of nuclear c-Myc protein was performed using antibodies against c-Myc and lamin A. Band intensities represent as the mean ± SD (within parenthesis) for the amount of c-Myc protein in comparison with the internal control lamin A. (**C**) Human PDAC HPAF-II cells were treated with DHA in the presence or absence of MG-132 in 10% FBS DMEM for 24 h. Cell survival was analyzed using MTT assay, as described in the Materials and Methods Section. Statistical significance is expressed as the mean ± SD (standard deviation) of three independent experiments. Different letters represent statistically significant differences within different subgroups (*p* < 0.05).

To explore the molecular mechanism of DHA, we further investigated its inhibitory effects on the STAT3/CAMKII signaling pathways in HPAF-II cells. As shown in Figure 3A, DHA potentiates the anti-cancer effects through a complementary reduction in phosphorylated EGFR, STAT3 and CAMKII proteins. Interestingly, DHA significantly induced the phosphorylation of c-Myc (at Thr 58 residue) protein in HPAF-II cells. We further verified the probable action and examine the effect of DHA on the stability of nuclear c-Myc protein in HPAF-II cells. As shown in Figure 3B, MG-132, a potent proteasome inhibitor, could reverse the expression of c-Myc protein upon the treatment of DHA in HPAF-II cells. Moreover, treatment of MG-132 could reverse cell death in DHA-stimulated HPAF-II cells (Figure 3C). These results suggested that DHA might suppress HG-mediated cell survival of HPAF-II cells through an inactivation of STAT3 and CAMKII signaling cascades, accompanied by modulating phosphorylation and expression levels of c-Myc protein in HPAF-II cells.

### 3.4. DHA Induced Induced Cell Death through an Increment of GSSG/GSH Ratio and Reactive Oxygen Species Level in HPAF-II Cells

**Figure 4:**
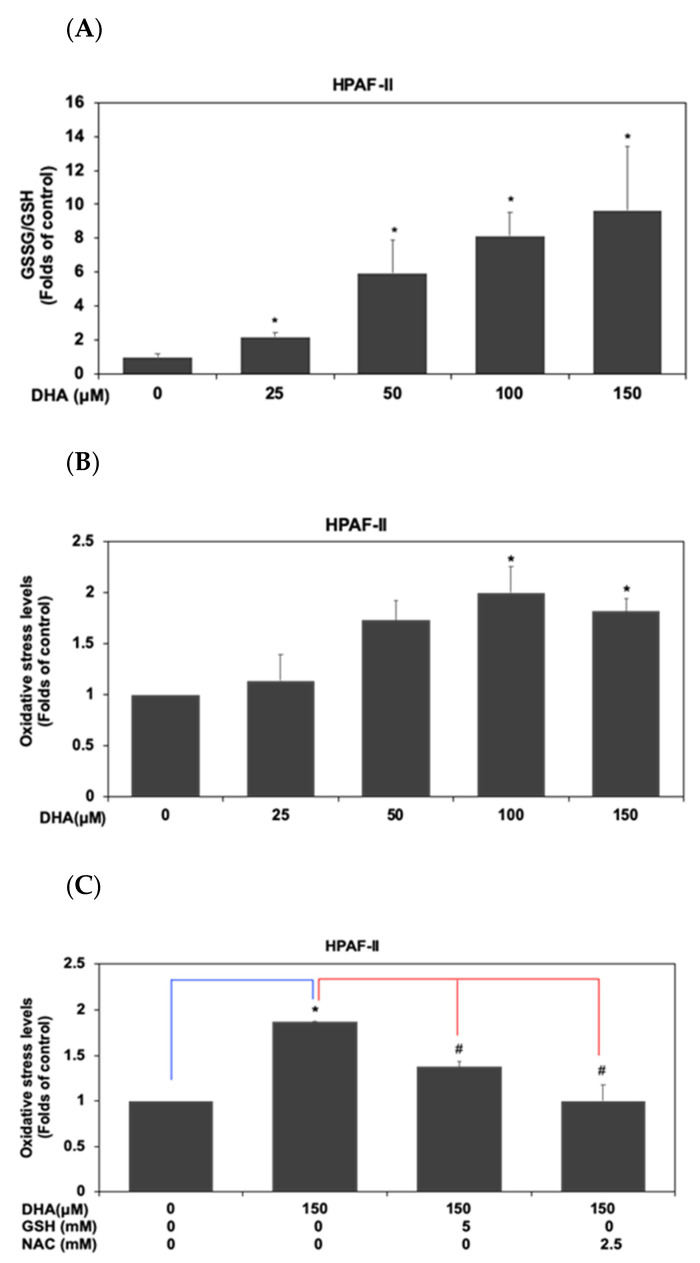

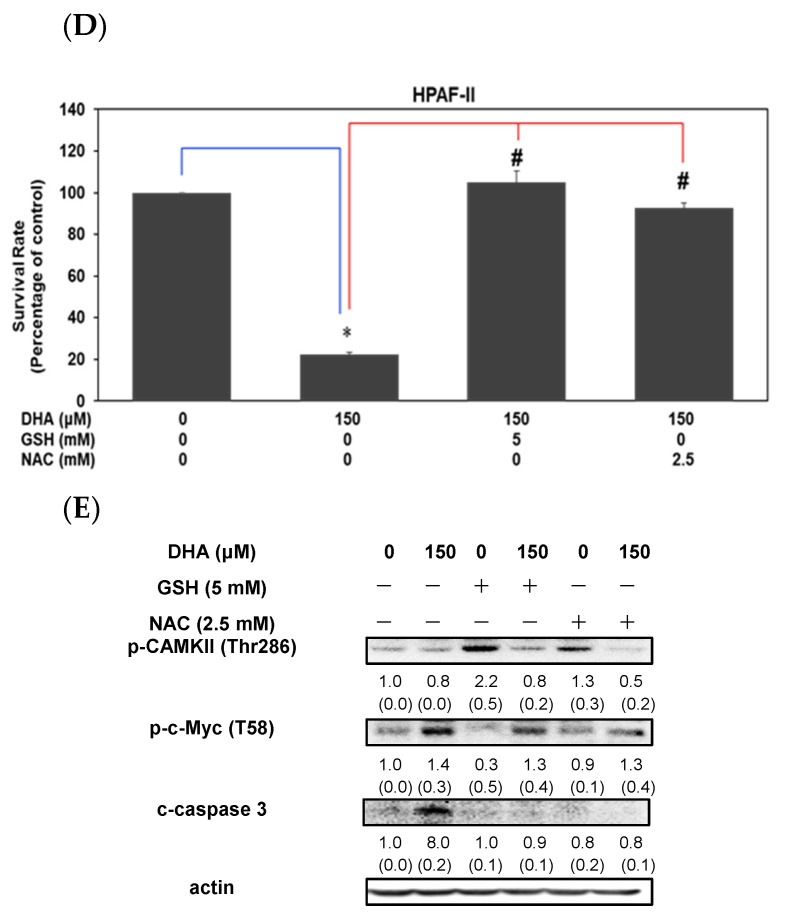
DHA induced cell death through an increment of GSSG/GSH ratio and reactive oxygen species level in HPAF-II cells. Human PDAC HPAF-II cells were treated with DHA at different concentrations in 10% FBS DMEM for 24 h. (**A**) Cellular levels of GSSG and GSH were measured by using LC- MS system as described in Material and Methods Section. (**B**) Cellular levels of ROS were measured using flow cytometry, according to the previous methods described in the Material and Methods Section. Human PDAC HPAF-II cells were treated with DHA in the presence or absence of GSH or NAC in 10% FBS DMEM for 24 h. (**C**) Cellular levels of free radicals were measured according to previous methods. (**D**) Cell survival was analyzed using MTT assay as described in the Materials and Methods Section. Statistical significance is expressed as the mean ± SD of three independent experiments. An asterisk (*) represents statistically significant differences in comparison with the control subgroup (*p* < 0.05). A pound (#) sign represents statistically significant differences in comparison with the DHA (150 μM)-treated subgroup (*p* < 0.05). (**E**) Human PDAC HPAF-II cells were treated with DHA in the presence or absence of GSH or NAC in 10% FBS DMEM for 24 h. Western blotting analysis of cytoplasmic proteins (at 24 h time point) was performed using antibodies against p-CAMKII (T286), p-c-Myc (T58), cleaved-caspase 3 (c-caspase3) and actin. Band intensities represent as the mean ± SD (within parenthesis) for the amount of each protein in comparison with actin.

Previous studies indicated that an increment of oxidative stress could lead to cell death of cancer cells [28]. Therefore, we further examined whether treatment of DHA could modulate oxidative stress levels in HPAF-II cells. As shown in Figure 4A, DHA significantly increased the GSSG/GSH ratio in HPAF-II cells. This result indicated that DHA could affect the oxidative stress in association with balance of oxidative and reduced forms of glutathione. Our results also demonstrated that DHA significantly enhanced reactive oxygen species (ROS) level in HPAF-II cells (Figure 4B) (*p* < 0.05). We further verified the effects of DHA on the induction of oxidative stress by using antioxidants such as GSH and NAC in this study. As shown in Figure 4C, treatment of GSH or NAC could reverse DHA-mediated increment of ROS level in HPAF-II cells. Moreover, GSH or NAC could reverse DHA-mediated cell death in HPAF-II cells (Figure 4D). These results indicated that DHA induced cell death, in part, through an increment of GSSG/GSH ratio and ROS level. We further examined the effects of GSH and NAC on DHA-mediated regulation of survival signaling pathways. As shown in Figure 4E, treatment of GSH or NAC alone could increase the phosphorylation of CAMKII protein in comparison with the control subgroup. However, treatment of GSH or NAC could not modulate the phosphorylation levels of CAMKII or c-Myc (T58) proteins in the presence of DHA. These results suggested that DHA modulated the phosphorylation levels of CAMKII or c-Myc (T58) proteins in an oxidant- independent manner. However, cotreatment of GSH or NAC could reverse the DHA-mediated expression of c-caspase 3 protein in comparison with the DHA-alone treated subgroup. These findings suggested that DHA might inhibit cell survival through an increment of oxidative stress levels.

### 3.5. DHA Inhibited Cell Survival through an Induction of Apoptosis in HPAF-II Cells

**Figure 5:**
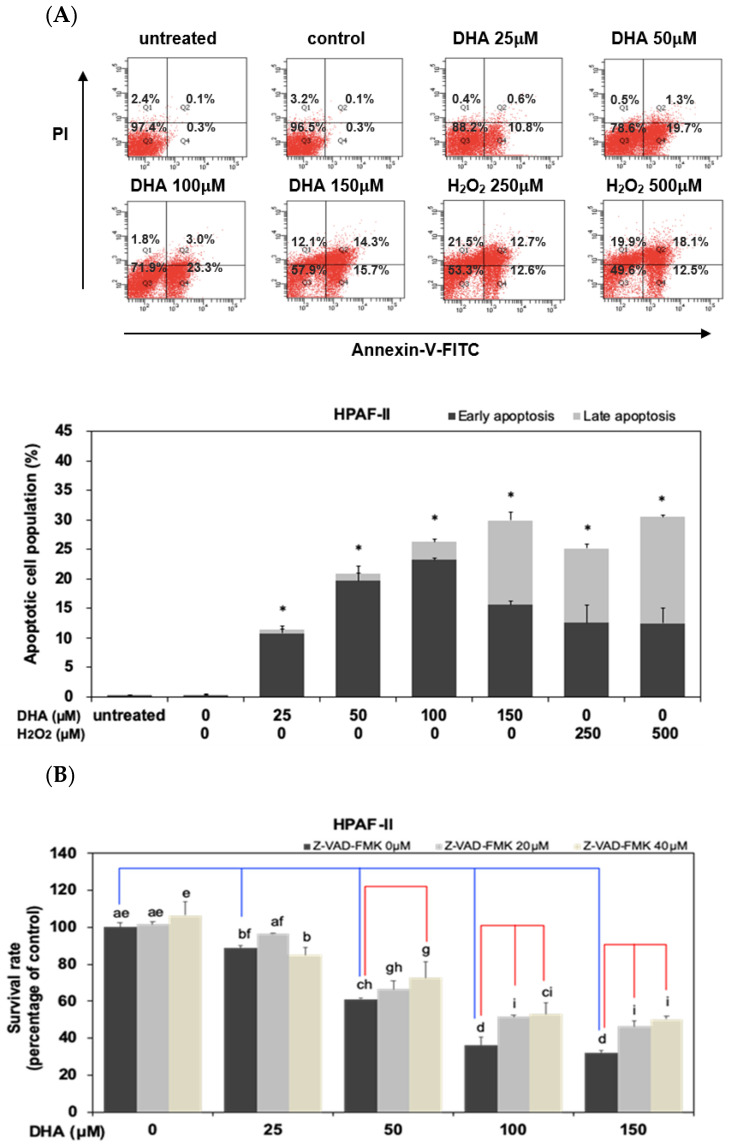

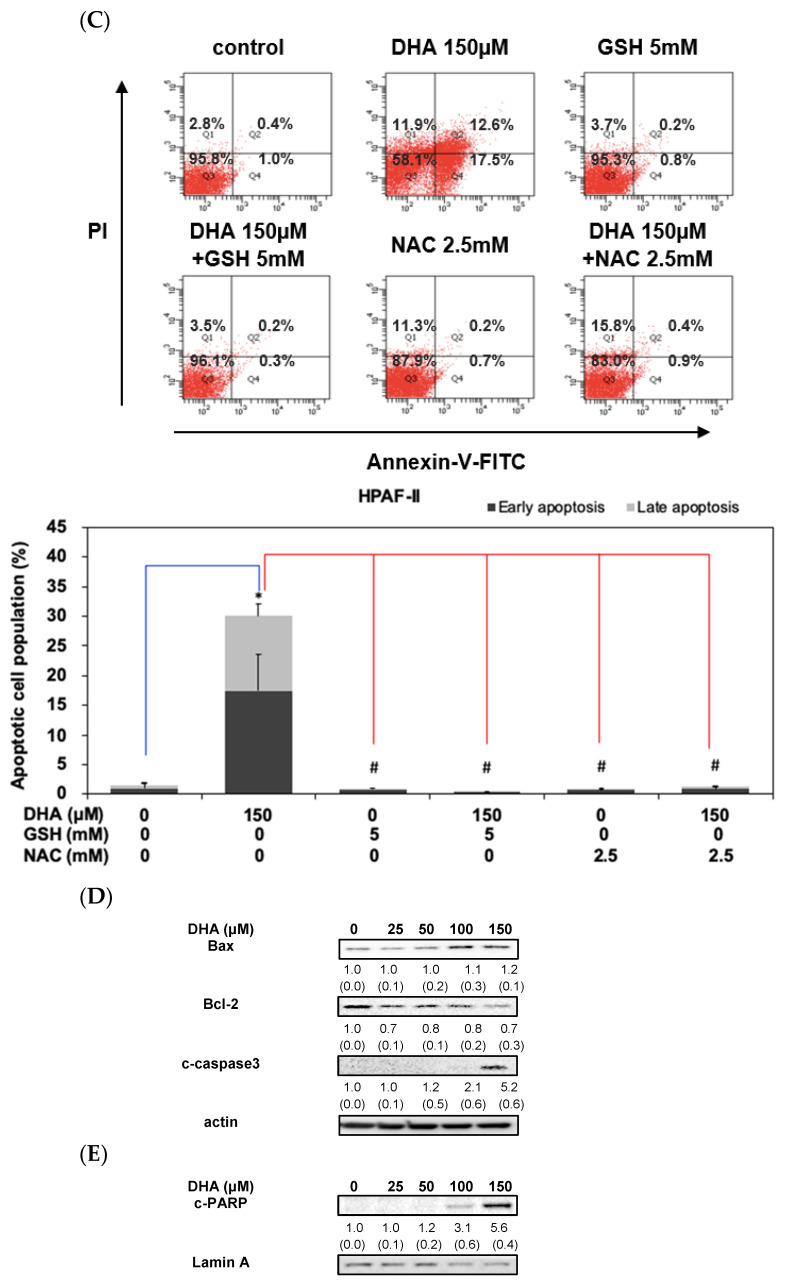
DHA inhibited cell survival through an induction of apoptosis in HPAF-II cells. Human PDAC HPAF-II cells were untreated or treated with DHA in 10% FBS DMEM for 24 h. H_2_O_2_ was used as a positive control(Supplementary Figure S1). (**A**) Analysis of cell apoptosis levels were performed using flow cytometry as described in Materials and Methods Section. The quantitative results of cellular apoptosis population were presented in the bottom panel. An asterisk (*) represent statistically significant differences in comparison with the control subgroup (*p* < 0.05). (**B**) Human PDAC HPAF-II cells were treated with DHA in 10% FBS DMEM in the presence or absence of Z-VAD-FMK (0, 20 and 40 μM) for 24 h. Cell survival rate was analyzed using MTT assay as described in Materials and Methods Section. Different letters represent statistically significant differences between different subgroups (*p* < 0.05). (**C**) Human PDAC HPAF-II cells were treated with GSH or NAC in the presence or absence of DHA. Analysis of cell apoptosis levels were performed using flow cytometry as described in Materials and Methods Section. The quantitative results of cellular apoptosis population were presented in the bottom panel. An asterisk (*) represents statistically significant differences in comparison with the control subgroup (*p* < 0.05). A pound (#) represents statistically significant differences in comparison with the DHA alone subgroup (*p* < 0.05). Western blotting analysis of cytoplasmic (**D**) and nuclear proteins (**E**) (at 24 h time point) was performed using antibodies against Bax, Bcl-2, c-caspase3, c-PARP, actin and lamin A. Band intensities are represented as the mean ± SD (within parenthesis) for the amount of cytoplasmic and nuclear proteins in comparison with internal control actin and lamin A proteins, respectively.

We further examined whether treatment of DHA could induce cell apoptosis in HPAF-II cells. As shown in Figure 5A, our results demonstrated that DHA significantly induced cell apoptosis in HPAF-II cells. To verify the probable mechanism of action, we further investigated whether DHA-mediated suppression of cell survival was through an induction of cell apoptosis in HPAF-II cells. As shown in Figure 5B, treatment of Z-VAD-FMK, a specific inhibitor of cell apoptosis, significantly reversed DHA- mediated cell death in HPAF-II cells. Moreover, treatment of GSH or NAC could reverse DHA-mediated cell apoptosis in HPAF-II cells (Figure 5C). DHA suppressed the expression of Bcl-2 protein in HPAF-II cells (Figure 5D). Moreover, DHA effectively enhanced the expression of apoptotic proteins such as Bax, c-caspase 3 and cleaved -PARP (c-PARP) in HPAF-II cells (Figure 5D–E). These results suggested that DHA inhibited cell survival, in part, through an induction of cell apoptosis in HPAF-II cells.

### 3.6. DHA Inhibited Nucleotide Synthesis in HPAF-II Cells

Our results indicated that DHA could inhibit cell proliferation of human PDAC HPAF-II cells in association with degradation and decreased expression of c-Myc protein. Several studies indicated that c-Myc protein was in involved in the expression of nucleotide synthesis and suggested that c-Myc is involved in the expression of nucleotide biosynthesis enzyme in cancer cells [29,30,31,32].

**Figure 6:**
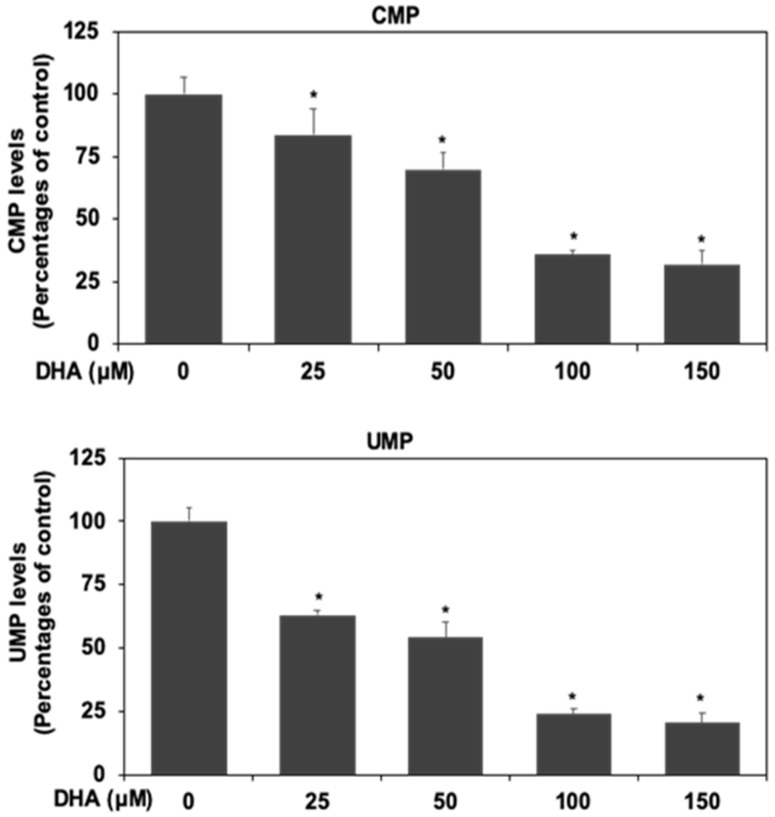
DHA inhibited nucleotide synthesis in HPAF-II cells. Human PDAC HPAF-II cells were treated with DHA at different concentrations in 10% FBS DMEM for 24 h. Cellular levels of CMP and UMP were measured by using LC- MS system as described in Material and Methods Section. An asterisk (*) represents statistically significant differences in comparison with the control subgroup (*p* < 0.05).

Thus, we further examined whether DHA could inhibit nucleotide synthesis, in association with the downregulation of c-Myc protein in HPAF-II cells. As shown in Figure 6, DHA suppressed intracellular levels of CMP and UMP in HPAF-II cells. These results suggested that DHA could inhibit nucleotide synthesis in association with a decreased expression of c-Myc protein in HPAF-II cells.

## 4. Discussion

Studies have demonstrated that human PDAC cells are highly additively to glucose metabolism [4]. N-3 PUFAs such as DHA have been suggested to be anti-inflammation agents [33]. Several studies demonstrated the inhibitory effects of DHA on the proliferation of pancreatic cancer cells [34,35]. Our previous study indicated that DHA could act as a potential agent for the treatment of pancreatic cancer [23]. However, the inhibitory effects of DHA on cell proliferation upon high glucose stimulation have not yet been well-studied. In the current study, our results demonstrated that high glucose significantly induced cell proliferation in comparison to the one at a normal glucose level (Figure 1A). These results indicate that the correlation between cell proliferation and glucose dependency were consistent with previous findings [9,36]. Therefore, we further investigated whether treatment of DHA could effectively inhibit cell proliferation of human PDAC cells upon high glucose stimulation. As shown in Figure 1, Our results showed that DHA inhibited cell proliferation through an induction of the cell cycle arrest at G_0_/G_1_ phase and decreased expression of cell cycle regulator proteins such as p-Rb, cyclin D1, cyclin E, cyclin A and E2F1 proteins (Figure 1B–D). DHA further inhibited the expression of c-Myc protein in human PDAC HPAF-II cells (Figure 1D). A previous study also indicated that hyperglycemia could induce the phosphorylation of STAT3 and the expression of c-Myc proteins in pancreatic cancer [9]. Our results further demonstrated that DHA could inhibit the expression of not only c-Myc, but also other cell cycle regulators such as p-Rb, cyclin D1, cyclin E, cyclin A and E2F1 proteins. A recent study indicated that DHA alone could not effectively inhibit the expression of c-Myc and cell proliferation in human colorectal cancer cells [37]. DHA could specifically act as an effective agent to inhibit the expression of c-Myc oncoprotein and cell proliferation in human PDAC cells, even under HG condition.

EGFR inhibition could abrogate STAT3 activation and increase survival rate in experimental animals [38]. Our results also showed that a blockade of EGFR activation could inhibit cell proliferation in human HPAF-II cells (Figure 2A). Another recent study demonstrated that CAMKII was involved in the stability and expression of c-Myc protein in cancer cells [21]. Therefore, we further examined the role of the STAT3 signaling pathway in the regulation of CAMKII and expression of c-Myc protein in human PDAC cells. Interestingly, treatment of ruxolitinib could inhibit the phosphorylation of CAMK-II and the expression of c-Myc oncoprotein in HPAF-II cells (Figure 2B,C). Moreover, blockade of STAT3 signaling pathway could enhance the phosphorylation of c-Myc protein (at Thr 58 residue) (Figure 2B). These results suggested at the pivotal role of STAT3 in the upregulation of CAMKII signaling molecule and the stability of c-Myc protein in HPAF-II cells. Our results suggested an interaction between the activation of STAT3/CAMKII signaling axis and the stability of c-Myc protein in HPAF-II cells upon HG stimulation although the detailed mechanism is still under investigation. This is the first evidence demonstrating the role of STAT3 signaling cascade in the regulation of CAMKII and its downstream c-Myc proteins in human PDAC cells. Therefore, we further examined the effects of DHA on the activation of survival cascades in HPAF-II cells. Our results showed that DHA effectively inhibited the phosphorylation of EGFR, STAT3 and CAMKII signaling proteins (Figure 3A). Moreover, DHA enhanced the phosphorylation of c-Myc protein (at Thr 58 residue) upon HG stimulation in HPAF-II cells (Figure 3A). Treatment of MG132, a specific inhibitor of proteasome, could further enhance the expression of c-Myc protein (Figure 3B) and cell survival (Figure 3C) under the stimulation of DHA in HPAF-II cells.

Several studies have indicated the association between oxidative stress and cell death in many types of cancer cells [39]. Therefore, we further examined whether DHA could inhibit cell proliferation through an increment of oxidative stress level and augmentation of cell apoptosis in HPAF-II cells. Our results demonstrated that DHA could significantly increase GSSG/GSH ratio in HPAF-II cells (Figure 4A). Moreover, treatment of DHA enhanced the intracellular ROS levels (Figure 4B,C). Suppression of intracellular ROS levels could reverse DHA-mediated cell death in HPAF-II cells (Figure 4D). Treatment of GSH or NAC could reverse DHA-mediated expression of c-caspase 3 protein. Our results further demonstrated that DHA effectively inhibited cell survival through an induction of cell apoptosis in HPAF-II cells (Figure 5A,B). Treatment of GSH or NAC could reverse DHA-mediated cell death in HPAF-II cells (Figure 5C). The mechanisms of action were through an increased expression of apoptotic proteins including Bax, c-caspase 3 and c-PARP (Figure 5D,E). These results suggested that DHA might induce cell death through an augmentation of GSSG/GSH ratio and increment of intracellular ROS levels. Previous studies suggested that c-Myc oncoprotein was involved in the gene expression of purine synthesis enzyme. Our results already demonstrated the inhibitory effects of DHA on the expression of c-Myc proteins. Our results further indicated that treatment of DHA could inhibit purine biosynthesis including decreased synthesis of CMP and UMP in HPAF-II cells (Figure 6).

**Figure 7:**
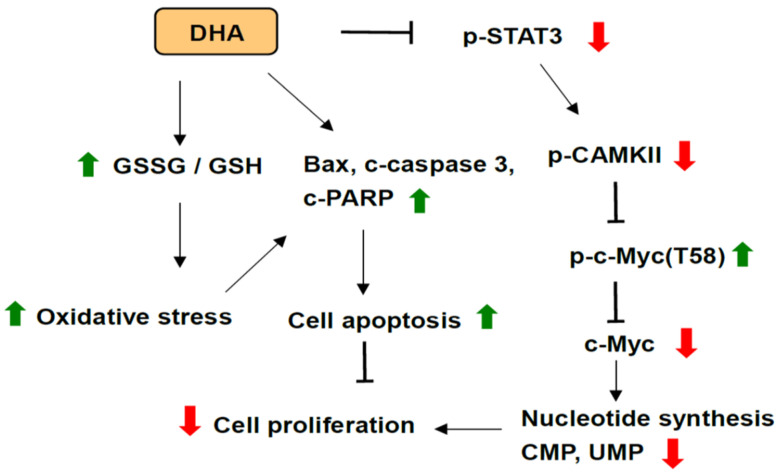
Proposed mechanisms of DHA-mediated suppression of STAT3/CAMKII signaling pathways, c-Myc expression and nucleotide synthesis as well as induction of apoptosis in human PDAC cells. Green arrows indicate an increased level. Red arrows indicate a decreased level. 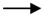: induction; 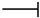: suppression.

In this study, our results demonstrated that DHA significantly inhibited cell proliferation through a blockade of STAT3/CAMKII signaling cascade, as well as instability and suppression of c-Myc protein accompanied with a decreased nucleotide biosynthesis in human PDAC cells upon high glucose stimulation. DHA further inhibited cell survival through an induction of cell apoptosis in association with increased GSSG/GSH ratio and intracellular ROS levels in human PDAC HPAF-II cells. The probable mechanisms are depicted in Figure 7.

## 5. Conclusions

In conclusion, we demonstrated a novel mechanism in which DHA could act as an effective agent to suppress cell proliferation of human PDAC HPAF-II cells upon HG stimulation.

## Data Availability

The data presented in this study are available in article.

## Supplementary Materials

The following are available online at https://www.mdpi.com/article/10.3390/antiox10111721/s1, Figure S1: the effects of DHA on smooth muscle A-10 cells which was used as a cell model of non-cancerous cells, Table S1: CC50 (IC50) Values (μM) and Selective Cytotoxicity Index (SCI) of pancreatic cancer cell Lines.
