# Supplementary material for: Docosahexaenoic Acid Inhibits Cell Proliferation through a Suppression of c-Myc Protein in Pancreatic Ductal Adenocarcinoma Cells
Source: Antioxidants (Basel). 2021 Oct 28;10(11):1721. doi: 10.3390/antiox10111721 (PMC8614909; doi:10.3390/antiox10111721)
Supplement: Supplementary file 1 [file antioxidants-10-01721-s001.zip › antioxidants-1404170-supplementary.pdf]

Supporting Information

Fig. 1D

	1	2
p-Rb (S795)	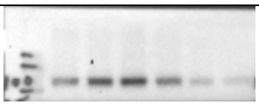	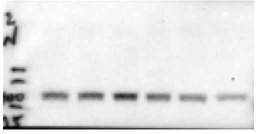
Rb	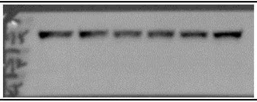	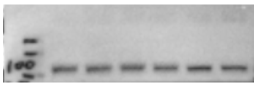
Cyclin D1	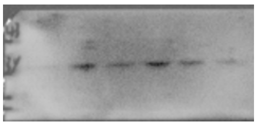	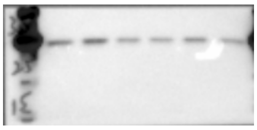
Cyclin E	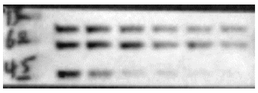	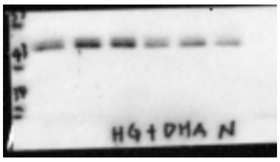
Cyclin A	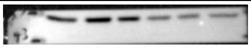	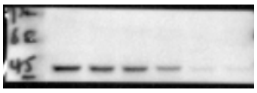
E2F1	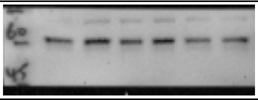	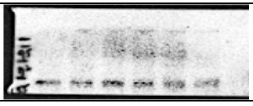
c-Myc	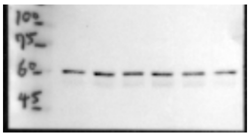	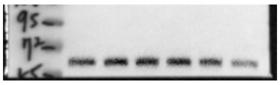
Lamin A	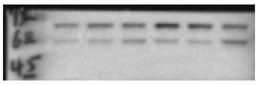	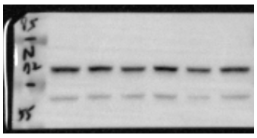

Fig. 2B

	1	2
p-CAMKII (T286)	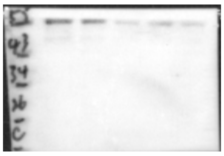	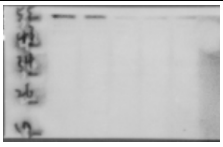
p-c-Myc (T58)	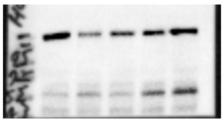	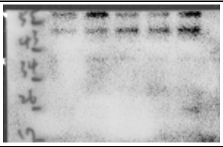
p-STAT3 (Y705)	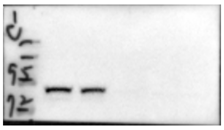	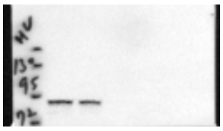
STAT3	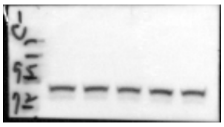	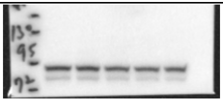
actin	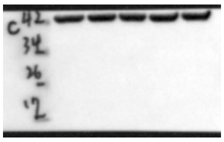	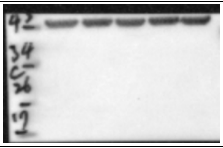

Fig. 2C

	1	2
**c-Myc**	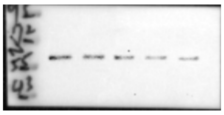	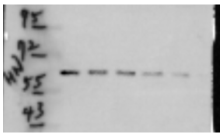
**lamin A**	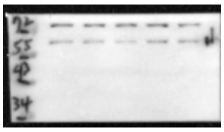	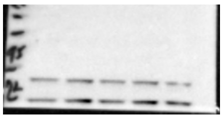

Fig. 3A

	1	2
**p-EGFR (Y1068)**	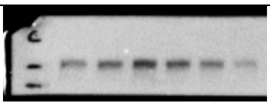	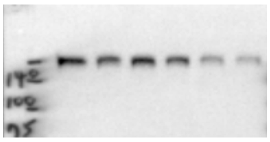
**p-STAT3 (Y705)**	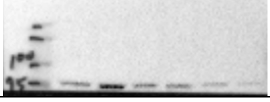	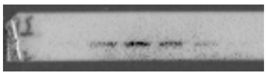
**t-STAT3**	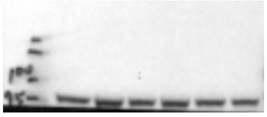	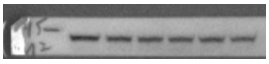
**p-CAMKII (T286)**	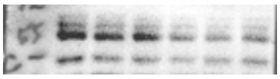	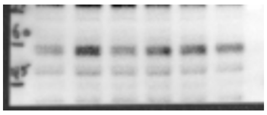
**p-c-Myc (T58)**	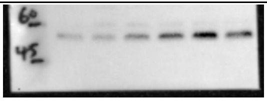	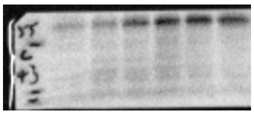
**actin**	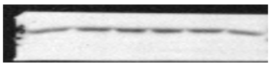	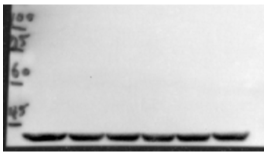

Fig. 3B

	1	2
**c-Myc**	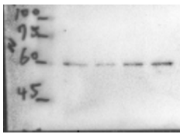	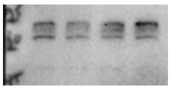
**Lamin A**	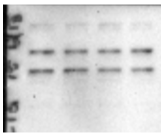	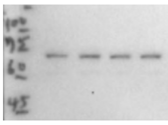

Fig. 4E

DHA (μM)	0 150 0 150 0 150	0 150 0 150 0 150
GSH (mM)	0 0 5 5 0 0	0 0 5 5 0 0
NAC (mM)	0 0 0 0 2.5 2.5	0 0 0 0 2.5 2.5
p-CAMKII (Thr286)	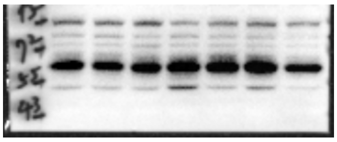	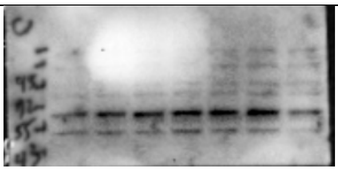
p-c-myc (T58)	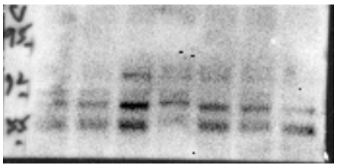	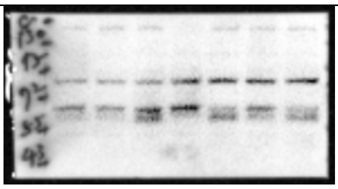
c-casapse3	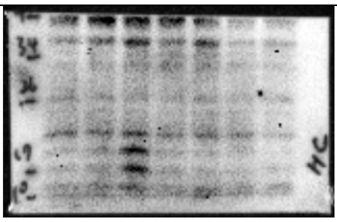	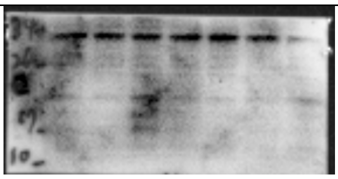
actin	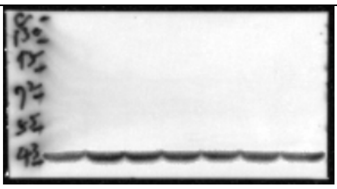	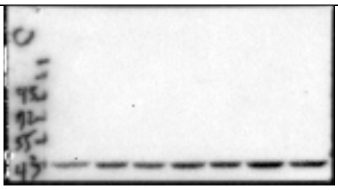

Fig. 5D

	1	2
Bax	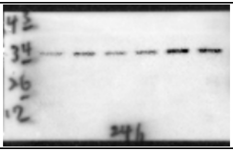	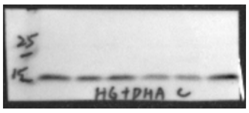
Bcl-2	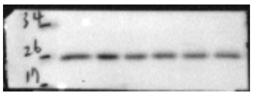	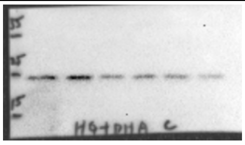
c-caspase3	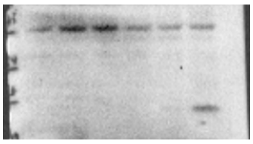	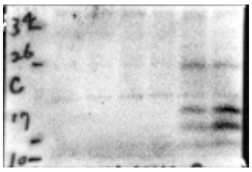

actin	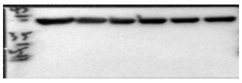	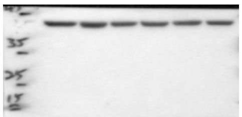

Fig. 5E

	1	2
c-PARP	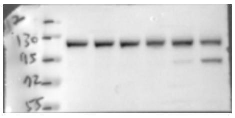	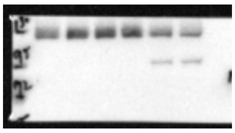
Lamin A	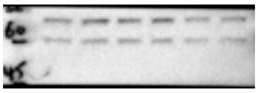	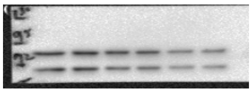

Figure S1.

(A)

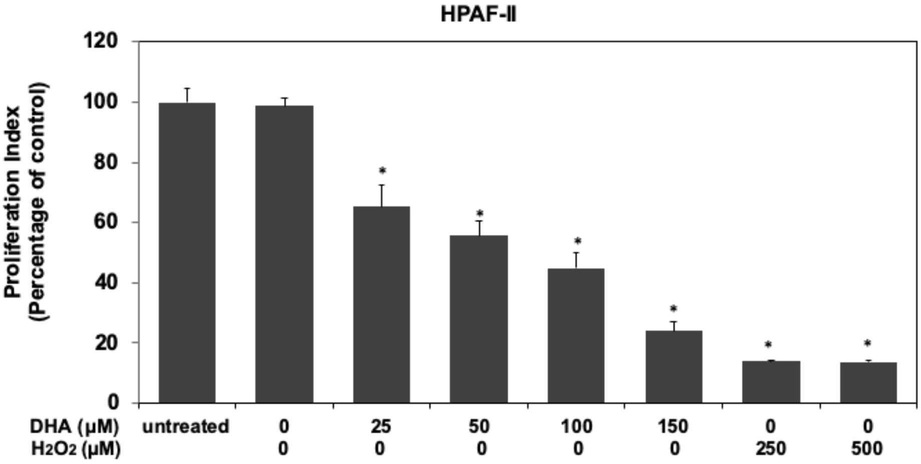

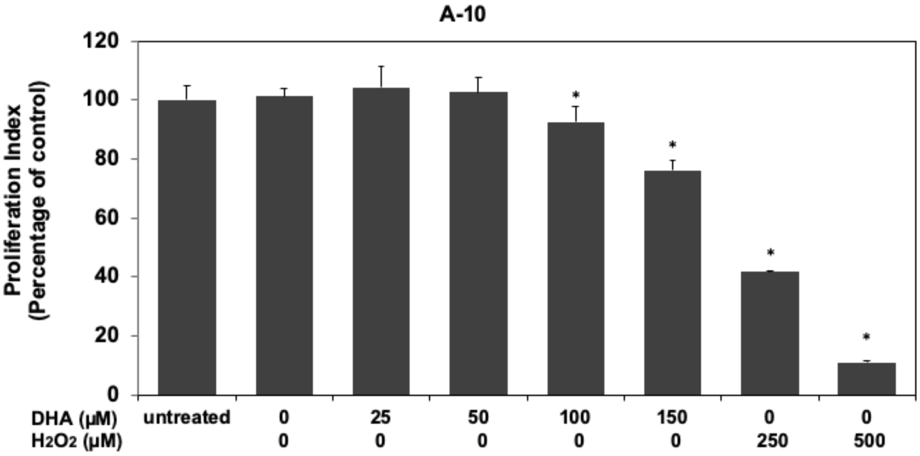

Table S1. CC_{50} (IC_{50}) Values (µM) and Selective Cytotoxicity Index (SCI) of pancreatic cancer cell Lines.

SCI = CC50 of non-cancerous cells/ CC50 of cancer cells.

**CC50 (µM)**				**SCI**		
**HPAF-II**	**CFPAC-1**	**MIA-Paca-2**	**A-10**	**HPAF-II**	**CFPAC-1**	**MIA-Paca-2**
80.71	399.65	309.65	280.66	3.48	0.70	0.91
SD=6.46	SD=75.77	SD=34.84	SD=26.01			
